# A Bioengineered In Vitro Model to Assess AAV-Based Gene Therapies for Cyclic GMP-Related Disorders

**DOI:** 10.3390/ijms23094538

**Published:** 2022-04-20

**Authors:** Marina Pavlou, Sabrina Babutzka, Stylianos Michalakis

**Affiliations:** 1 Department of Biological Structure, University of Washington, Seattle, WA 98195, USA; mpavlou@uw.edu; 2 Department of Ophthalmology, University Hospital, LMU Munich, 80336 Munich, Germany; sabrina.babutzka@med.uni-muenchen.de

**Keywords:** retina, AAV, cGMP, in vitro potency assay, biological activity, transgene expression

## Abstract

The emergence of efficient viral vectors derived from adeno-associated viruses (AAV) has led many groups to develop gene therapies for inherited monogenic diseases, such as retinal dystrophies. To evaluate the potency of new gene therapy vectors in a preclinical context, it is common to use animal models, such as gene-deficient or mutant animal models of a given human disease, and then assess vision restoration with functional or behavioral assays. While such animal models are invaluable to the preclinical testing process, they cannot be readily used as batch release tests during manufacturing or to validate biological activity at later stages of development. There is therefore a need for rapid and reliable in vitro models that can determine whether therapeutic vectors have delivered their cargo gene, and more importantly, whether this has resulted in the intended biological activity. Given our previous experience, we chose CNGA3-linked achromatopsia to develop a cell-based system to verify biological activity of AAV vectors designed to deliver a healthy *CNGA3* gene copy into human cone photoreceptors. Our system is based on an immortalized cell line with high susceptibility to AAV transduction, i.e., HeLa cells, which we engineered to express a fungal rhodopsin guanylyl cyclase (RhGC) from *Blastocladiella emersonii* and a sensitive genetically encoded calcium indicator (GECI) under the control of a tetracycline operator. Using this system, we were able to confirm and quantify the function of the ion channel encoded by AAV/CNGA3 and differentiate between AAV vector potencies with a simple fluorometric assay. Finally, we show that this approach can be readily adapted for the assessment of phosphodiesterase function.

## 1. Introduction

Monogenic retinal diseases are ideal candidates for gene therapy as they are both genetically and phenotypically well characterized, with single-gene supplementations having a therapeutic impact. The emergence of recombinant adeno-associated viral (AAV) vectors, with high tropism for retinal cells, has made it possible to locally deliver gene therapies with intraocular injections. This has sparked numerous efforts to develop new and optimized AAV-based gene therapies to treat the plethora of blinding monogenic diseases. Throughout the development of new gene therapies, there is a need for informative in vitro assays that can be performed rapidly and cost-effectively before in vivo testing to facilitate the evaluation of different promoters, codon-optimized genes, and optimized regulatory genetic elements. In addition, functional potency assays are required for quality control and batch release testing during good manufacturing practice (GMP) production and for stability studies of a final gene therapy drug product.

A monogenic disorder for which five gene therapy candidates are currently being tested in clinical trials is achromatopsia. Achromatopsia is characterized by severe impairment of visual acuity and color vision as well as photophobia and pendular nystagmus. To date, six genes (*ATF6A*, *CNGA3*, *CNGB3*, *GNAT2*, *PDE6C*, and *PDE6H*) are known to cause achromatopsia, with mutations in *CNGA3* and *CNGB3* responsible for more than 90% of cases [1]. *CNGA3* and *CNGB3* encode the alpha and beta subunits of the cone cyclic nucleotide-gated (CNG) channel that mediates the depolarizing Na^+^/Ca^2+^ “dark” current in cone photoreceptor outer segments [2]. Previously, the potency of gene therapy vectors for the treatment of *CNGA3*- or *CNGB3*-linked achromatopsia was assessed after subretinal or intravitreal injection of the gene therapy vector in gene-deficient animal models (i.e., mouse or dog) [3,4,5,6,7,8,9,10]. After an incubation period of several weeks to months, the treatment effect was determined by gene expression analyses to assess *CNGA3* or *CNGB3* transgene expression and vision-guided behavior and/or electroretinography (ERG) to indirectly assess the biological activity of the expressed CNGA3/CNGB3 cGMP-gated ion channel. Such in vivo potency testing of gene therapy vectors has low throughput and can only be performed by trained personnel in specialized animal facilities equipped for ophthalmological procedures and examinations. This also depends on the availability of a sufficient number of animals and can take several weeks or months to establish therapeutic efficacy. As such, a rapid and simple in vitro system that could functionally evaluate new viral vectors would substantially streamline potency testing of gene therapy vectors while reducing costs.

To this end, we engineered a cell-based system that can indirectly track the presence of a functional AAV-borne CNG channel using a simple fluorometric assay. We show that this system can be used to reliably assess the biological function of AAV vectors expressing CNGA3 and, with some adaptation, vectors expressing PDE6A.

## 2. Results

### 2.1. The Concept of the Cell-Based In Vitro Potency System

**Figure 1:**
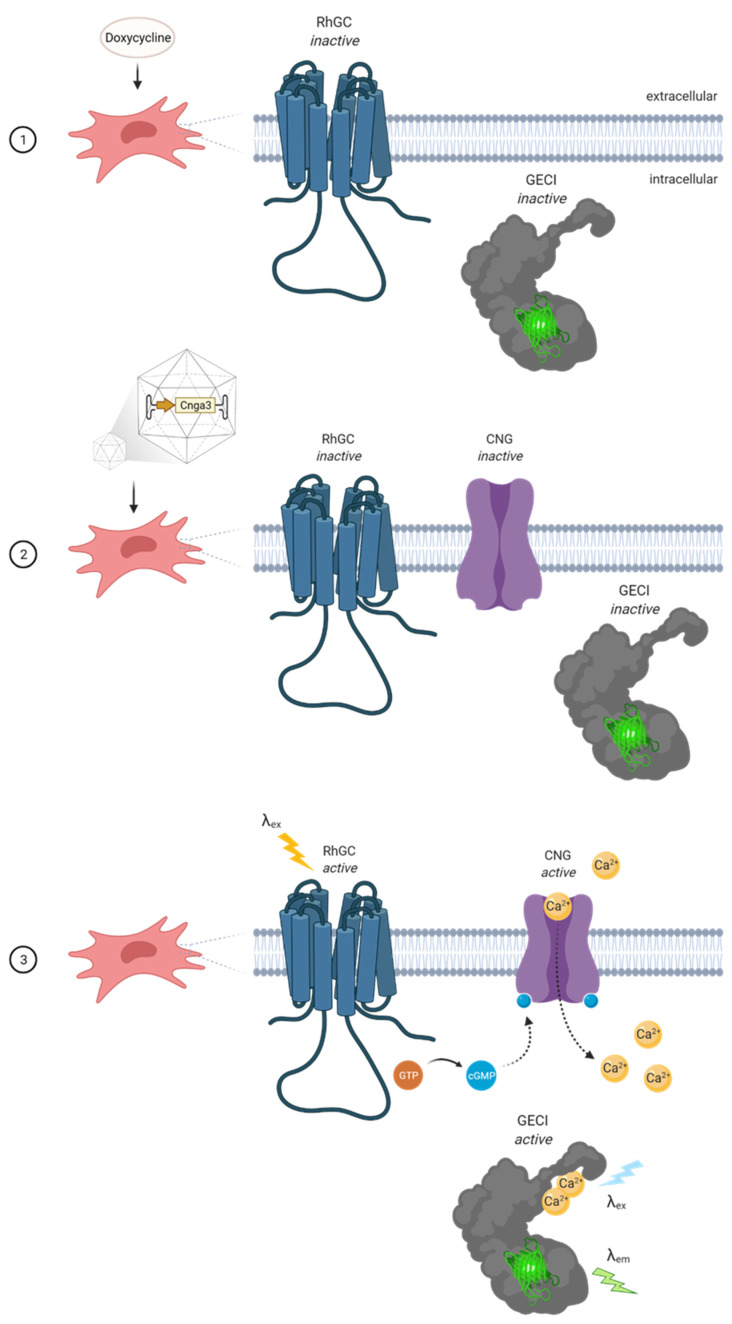
Schematic diagram showing the stepwise activation of the bioengineered cell system where (1) doxycycline induces the expression of rhodopsin guanylyl cyclase (RhGC) and genetically encoded calcium indicator (GECI) that remain inactive. (2) Transduction of the cells with AAV/CNGA3 leads to the expression of a homotetrameric cyclic nucleotide-gated (CNG) channel that remains closed in the absence of the ligand cyclic guanosine monophosphate (cGMP). (3) Illumination of cells at the RhGC excitation wavelength (λex) leads to cGMP production that binds and opens the CNG channel leading to a Ca^2+^ influx. When illuminated at λex, Ca^2+^-bound GECI emits a specific fluorescent signal (λem) that can be quantified by a fluorometric plate reader.

With the aim to develop a cell-based system that can detect CNG channel function, we genetically engineered HeLa cells to express an inducible protein cascade that forms a non-ratiometric reporter for cGMP-gated ion influx. HeLa cells were chosen because of their high susceptibility for AAV transduction [11] and universal accessibility. We engineered the cells using the piggyBac transposon system [12] to stably express the fungal rhodopsin guanylyl cyclase (RhGC) from *Blastocladiella emersonii* [13] and a sensitive genetically encoded calcium indicator (GECI), either GCaMP6s [14], jRCaMP1a or jRCaMP1b [15]. We placed these genes under the control of a tetracycline ON (TetON) system [16,17] in order to induce their expression in a controlled manner (see Table 1 for qPCR primers used to verify expression). As such, only cells exposed to the tetracycline analogue doxycycline would express RhGC and the GECI. HeLa cells do not naturally express CNG channels. We therefore hypothesized that these cells could be used to test the potency of novel AAV vectors with a transgene cassette encoding cGMP-gated ion channel subunits, such as CNGA3. Once transduced with an AAV/CNGA3 vector, and in the presence of doxycycline, the engineered cells would simultaneously express the light-controlled RhGC, the Ca^2+^ sensor GECI, and a homomeric cGMP-gated Ca^2+^ channel CNGA3. When the cells are illuminated at an excitation wavelength (λex) of 500–560 nm, RhGC would generate cGMP [13] that would bind and open the CNGA3 channels on the plasma membrane. This would allow Ca^2+^ to enter the cell and bind to the GECI, which would fluorescently indicate the change in intracellular Ca^2+^ content. This would indirectly confirm the presence of a functional vector-borne CNGA3 channel (Figure 1).

### 2.2. Validation of Basic Functions

In order to test whether the three selected GECIs would detect Ca^2+^ under physiological conditions, the stable cell lines were exposed to increasing concentrations of doxycycline, i.e., 0.25, 0.5 and 1 μg/mL. After 48 h incubation, the Ca^2+^-free medium was replaced with Ca^2+^-containing medium (HBSS^+CaCl_2_^) and the cells were imaged using a fluorescence microscope with filters for GFP (λex 488, λem 510) and Texas Red (λex 596, λem 615). The signal obtained was comparable for all concentrations (Figure S1A), and therefore cells were treated with 0.5 μg/mL doxycycline for all subsequent experiments. As a positive control for GECI fluorescence when bound to Ca^2+^, the cells were treated with 4 μM ionomycin for 5 min, which led to the partial disruption of their cell membrane and an uncontrolled influx of Ca^2+^. By comparing the fluorescent images before and after ionomycin treatment, it was evident that the signal from GCaMP6s (Figure S1A) and jRCaMP1a (Figure S1B) differed the most between conditions, whereas that from jRCaMP1b (Figure S1C) did not. Therefore, the cells with jRCaMP1b were not used for further experiments because this poor sensitivity would fail to detect any CNG-borne changes in intracellular Ca^2+^.

**Figure 2:**
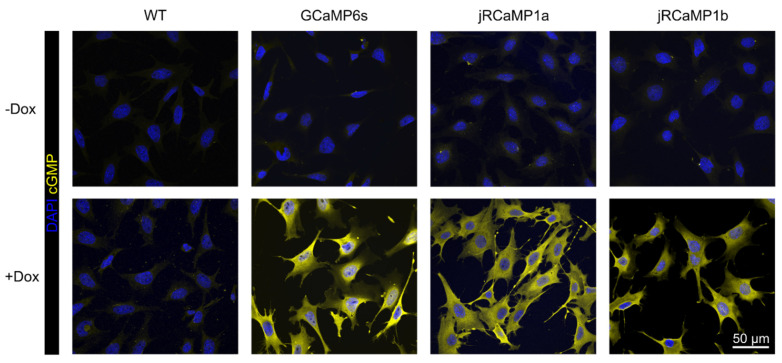
Validation of light-inducible production of cGMP by RhGC in the three engineered HeLa cell lines compared to the parental wild-type (WT) HeLa cells. Confocal images of cells cultured with or without doxycycline, illuminated at the RhGC activation wavelength and then immunolabeled for cGMP (yellow). Nuclei were stained with DAPI (blue).

The light-inducible function of RhGC was validated in all three stable cell lines. Cells were incubated with 0.5 μg/mL doxycycline for 48 h and then the media was replaced with DPBS^−CaCl_2_^. The cells were then illuminated in a warmed (37 °C) plate reader at 500–560 nm in 10 nm intervals to activate the RhGC. Once illuminated, the cells were immediately fixed and immunolabeled for cGMP using an antibody that detects paraformaldehyde-fixed cGMP. Confocal imaging showed that only the engineered cells stained strongly for cGMP in the presence of doxycycline, whereas the parental wild-type cells did not. Furthermore, none of the cell lines generated a detectable cGMP signal in the absence of doxycycline (Figure 2), thereby confirming that the TetON-dependent RhGC is tightly regulated and induces cGMP production only after illumination.

**Figure 3:**
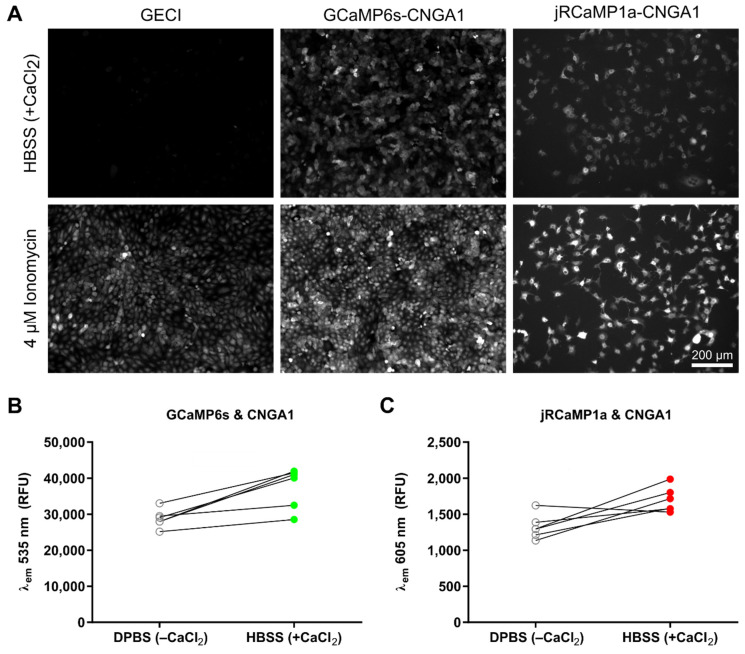
Positive control series with stably integrated CNG channel to confirm that the binary reporter system works in the presence of all three constituent proteins, i.e., RhGC, CNG and GECI. (**A**) Fluorescence images of GECI signal obtained from engineered HeLa cells expressing either RhGC-GECI or RhGC-GECI-CNGA1, in the presence of high extracellular Ca^2+^ (HBSS^+CaCl_2_^) versus positive control signal, where cells are permeabilized with a 4 μM ionomycin solution. (**B**) Quantification of relative fluorescence units (RFU) at λem 535 nm of engineered HeLa cells expressing RhGC-GCaMP6s-CNGA1 in the absence of extracellular Ca^2+^ (DPBS^−CaCl_2_^) versus high extracellular Ca2+ (HBSS^+CaCl_2_^). (**C**) Quantification of RFU at λem 605 nm of engineered HeLa cells expressing RhGC-jRCaMP1a-CNGA1 in the absence of extracellular Ca^2+^ (DPBS^−CaCl_2_^) versus high extracellular Ca^2+^ (HBSS^+CaCl_2_^).

It was important to determine whether the components of this cell-based reporter could work together in the presence of a CNG channel. Using a new piggyBac vector, the HeLa cells were further modified to express the *CNGA1* gene under the same TetON system in addition to RhGC-GCaMP6s or RhGC-jRCaMP1a. This means that all three proteins would be expressed after exposing the cells to doxycycline and the Ca^2+^ flux after RhGC illumination could be tested by measuring changes in the GECI signal on a plate reader. The baseline signal for GCaMP6s and jRCaMP1a was measured using the maximum sensitivity settings. Subsequently, the extracellular Ca^2+^ concentration was increased by replacing DPBS^−CaCl_2_^ with HBSS^+CaCl_2_^, and the test signal for both GECIs was again measured. Finally, using a microinjector as part of the plate reader, ionomycin was added to the cells to a final 4 μM concentration, and the maximum GECI signal was recorded. Fluorescence imaging alongside plate reader recordings revealed that the cells expressing RhGC-GECI-CNGA1 gave a strong signal in the presence of extracellular Ca^2+^, as opposed to RhGC-GECI alone (Figure 3A). This was reflected in the signal quantification, where both GCaMP6s (Figure 3B) and jRCaMP1a (Figure 3C) signal significantly increased as a result of CNGA1-dependent Ca^2+^ influx.

### 2.3. Testing AAV Vectors with CNGA3 Cargo

After validating our cell-based reporter system, we set out to test whether the two engineered cell lines RhGC-GCaMP6s and RhGC-jRCaMP1a could detect AAV-borne CNG activity. Both lines were treated with doxycycline and transduced with an AAV/CNGA3 vector for 48 h at multiplicity of infection (MOI) 10^4^ and 10^5^. As before, the medium was replaced with DPBS^−CaCl_2_^, and the cells were illuminated in a heated (37 °C) plate reader at 500–560 nm in 10 nm intervals to induce cGMP production. The baseline signal for GCaMP6s and jRCaMP1a was measured and then DPBS^−CaCl_2_^ was replaced by HBSS^+CaCl_2_^. The test signal for both GECIs was measured, and finally, ionomycin was added to record the maximum GECI signal in the transduced cells. Fluorescence imaging acquired alongside the plate reader recordings showed that there was a detectable jRCaMP1a signal already in the untreated cells (Figure S2A), which was also seen in Figure S1B, and the signal remained the same for both MOIs (Figure S2A). Instead, the GCaMP6s signal was restricted to the AAV-treated cells, showing no qualitative difference between the two MOIs (Figure S2A). The quantification of jRCaMP1a showed that, although there was a higher signal in the presence of extracellular calcium (HBSS^+CaCl_2_^), this was not significantly different to the positive control condition (Figure S2B). This was inconsistent with the fluorescence imaging of the cells, suggesting that jRCaMP1a could not be used confidently across signal detection methods. Unlike jRCaMP1a, the signal obtained from GCaMP6s was consistent across methods, with both plate reader quantification and fluorescent imaging of the transduced cells matching (Figure S2C). This confirmed that the engineered HeLa cells with RhGC-GCaMP6s could detect AAV-borne CNGA3 channel function

**Figure 4:**
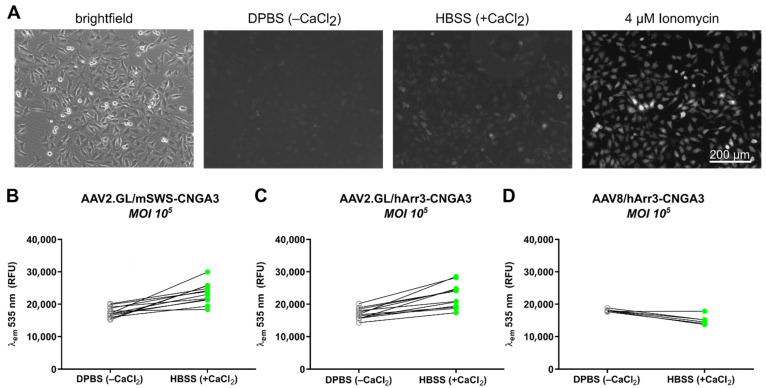
Discrimination of vector potencies using the optimal engineered HeLa cells expressing RhGC-GCaMP6s. (**A**) Representative fluorescence images of GCaMP6s signal after transduction with AAV2.GL/hArr3-CNGA3 in three conditions: in the absence of extracellular Ca^2+^ (DPBS^−^^CaCl_2_^), in high extracellular Ca^2+^ (HBSS^+CaCl_2_^), and positive control signal where cells are permeabilized with a 4 μM ionomycin solution. (**B**) Quantification of relative fluorescence units (RFU) at λem 535 nm cells transduced with AAV2.GL/mSWS-CNGA3 in the absence of extracellular Ca^2+^ (DPBS^−^^CaCl_2_^) versus high extracellular Ca^2+^ (HBSS^+CaCl_2_^). (**C**,**D**) As in (**B**) for cells transduced with AAV2.GL/hArr3-CNGA3 and AAV8/hArr3-CNGA3, respectively.

To assess whether our cell-based reporter could discriminate between vector potencies, three different AAV vectors expressing mouse or human CNGA3 were tested using the same experimental set-up as above. Vectors carried either a human hArr3-CNGA3 or a mouse mSWS-CNGA3 expression cassette and were packaged with either the engineered AAV2.GL [10] or the conventional AAV8 capsid. All vectors were used at an MOI of 10^5^, which was sufficient to increase the detected GCaMP6s signal in the presence of high extracellular Ca^2+^ (Figure 4A). Quantitative results confirmed the presence of a functional vector-borne CNG channel after transducing cells with AAV2.GL/mSWS-CNGA3 (Figure 4B) and AAV2.GL/hArr3-CNGA3 (Figure 4C), but not AAV8/hArr3-CNGA3 (Figure 4D), indicating that the vectors with AAV2.GL were more potent.

### 2.4. Combined AAV Vector Testing with CNGA3 and PDE6A Cargo

**Figure 5:**
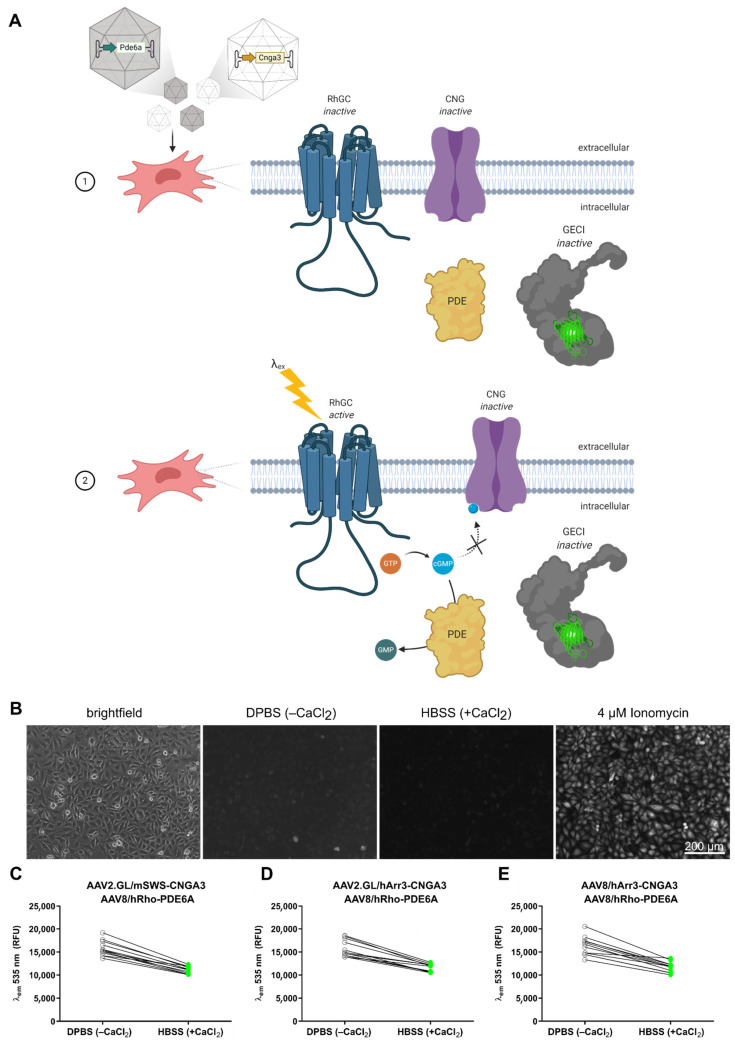
Adaptation of cell-reporter system to test AAVs with PDE6A cargo. (**A**) Schematic diagram outlining the predicted interaction of the cell-reporter system when (1) cells are co-transduced with AAV/CNGA3 and AAV/ PDE6A, leading to the expression of both the homotetrameric CNG channel and a partial PDE protein. RhGC illumination (2) leads to cGMP production that, instead of binding and activating the CNG, it becomes hydrolyzed by the PDE, thereby preventing Ca^2+^ influx and GECI signal. (**B**) Representative fluorescence images of GCaMP6s signal obtained from engineered HeLa cells after transduction with CNGA3- and PDE6A-expressing vectors in three conditions: in the absence of extracellular Ca^2+^ (DPBS^−^^CaCl_2_^), in high extracellular Ca^2+^ (HBSS^+CaCl_2_^), and positive control signal where cells are permeabilized with a 4 μM ionomycin solution. (**C**) Quantification of relative fluorescence units (RFU) at λem 535 nm of engineered HeLa cells co-transduced with AAV2.GL/mSWS-CNGA3 and AAV8/hRho-PDE6A in the absence of extracellular Ca^2+^ (DPBS^−^^CaCl_2_^) versus high extracellular Ca^2+^ (HBSS^+CaCl_2_^). (**D**,**E**) As in (**C**) for cells co-transduced with AAV2.GL/hArr3-CNGA3 & AAV8/hRho-PDE6A or AAV8/hArr3-CNGA3 & AAV8/hRho-PDE6A, respectively.

To broaden the applicability of this in vitro model, we simultaneously transduced the engineered HeLa cells with AAV vectors that carried two cGMP effector molecules with opposing effects: the previously used CNGA3 channel, which increases intracellular Ca^2+^ levels in the presence of cGMP, and the phosphodiesterase 6 alpha subunit (PDE6A), which degrades cGMP and thus counteracts the CNG-mediated cation influx, leading to a decrease in intracellular Ca^2+^ (Figure 5A). In our previous experiments, we observed a basal Ca^2+^ level that led to measurable GCaMP6s signal even in the DPBS^−^^CaCl_2_^ solution (Figure 4). We therefore hypothesized that if both proteins (CNGA3 and PDE6A) were expressed, the cGMP required for CNG-dependent Ca^2+^ influx would become hydrolyzed by the PDE6, leading to a further decrease in the GCaMP6s signal (Figure 5A). We co-transduced cells with AAV vectors encoding both CNGA3 and PDE6A at an MOI 10^4^, which led to detectable levels of both proteins, as confirmed via ICC (Figure S3). Transduction with individual vectors (Figure S3A) led to comparable protein expression levels, as seen in cells co-transduced with both AAVs (Figure S3B). Indeed, representative fluorescent images showed that the minimal baseline GCaMP6s signal was diminished in cells co-transduced with CNGA3 and PDE6A after switching to a high extracellular Ca^2+^ solution (Figure 5B). This phenomenon was reflected also in the plate reader signal quantification, showing that all three vector combinations of simultaneous CNGA3 and PDE6A delivery resulted in decreased GCaMP6s signal (Figure 5C–E).

## 3. Discussion

During drug development, a series of preclinical tests are performed to verify the safety and efficacy of a new therapeutic agent before being cleared for human clinical trials. The requirements for preclinical testing are often defined by the disease the new drug is intended for and the drug’s pharmacological properties. In the context of viral vector-based gene therapy drugs, new vectors must be tested both in vitro and in vivo [18]. The use of in vitro systems, such as mammalian cell culture, generates proof-of-principle data that help sieve through large libraries of constructs ahead of more lengthy and costly experiments in vivo using relevant animal models. Unfortunately, not all diseases for which new gene therapies are developed have a relevant animal model that mirrors the human pathophysiology. Even in cases where such a model exists, in vivo testing of new gene therapies takes months before a functional evaluation can be made. During the development and optimization of new gene therapies, it is often necessary to repeatedly assess vector potency and functionality, making it exponentially harder to incorporate in vivo assays at all the qualitative checkpoints. This is where simple in vitro assays that can evaluate vector function can be of significant value.

For the purpose of assessing AAV vector potency and biological function, an immortalized cell line with high AAV transduction susceptibility was used, namely, HeLa cells [11]. This in vitro model was designed to assess vectors with a transgene cassette expressing the CNGA3 subunit of the human cone-specific CNG channel, which would be used to treat achromatopsia patients with mutations in this gene. The primary aim was to confirm the formation of a functional ion conducting CNG channel after AAV delivery, and therefore required constituents that would interact with the channel to form a reporter system. These constituents were cGMP, as CNG channels in the retina are gated by the ligand cGMP [19], and a quantifiable signal that would report ion channel function, i.e., cation influx. To this end, the piggyBac transposon system [12] was used to integrate a light-inducible RhGC [13] and a GECI into the cells’ genome, under the control of a TetON system [16,17]. The resulting cells were validated for their ability to produce cGMP after RhGC illumination (Figure 2) and their inducible GECI expression after exposure to doxycycline (Figure S1). From the three GECIs tested, i.e., GCaMP6s, jRCaMP1a and jRCaMP1b, only GCaMP6s proved to be reliable across signal detection methods (Figures S1 and S2). Indeed, GCaMP6s has the highest calcium affinity from the other GCaMP6 family members [14] and has been deemed optimal for high-throughput assays [20].

A positive control study was performed, where the rod-specific CNGA1 subunit was endogenously overexpressed via the same TetON system, to confirm that the three components RhGC, CNG and GECI would interact to form a fluorescence reporter for Ca^2+^ influx (Figure 3). It was sufficient to introduce the alpha subunit of CNG, as these can form functional homomeric ion channels, unlike the beta subunits when expressed alone [21]. The engineered cell line was successfully used to discriminate CNGA3-carrying vectors with distinct potencies (Figure 4), as well as demonstrate the applicability of this system to test vectors with a PDE6A-expressing cassette in the presence of CNGA3 (Figure 5). Note that transgene cassettes with photoreceptor-specific promoters were able to drive gene expression in HeLa cells, where such promoters should be inactive. Promoter activation *in trans* has been reported in transfection experiments [22], indicating the possibility that, at high enough MOIs, the interaction between the AAV ssDNA and HeLa genome can trigger sufficient gene expression under an otherwise less active promoter. Another important consideration is that single-protein subunits were delivered. Though it is known that CNG alpha subunits (with the exception of CNGA4) can form homomeric channels [19,21], it was not clear whether the rod PDE6A subunit would suffice to perform cGMP hydrolysis. The native rod PDE6 enzyme is thought to consist of two catalytic subunits PDE6A and PDE6B, and two inhibitory subunits PDE6G [23]. Using our in vitro model, the hydrolysis of cGMP was measured indirectly by the decrease in CNG-mediated GCaMP6s signal (Figure 5B,C). The fact that the GCaMP6s signal decreased below the baseline (Figure 5C) was likely an effect of time rather than the change in extracellular solution. It is possible that the gradually decreasing GCaMP6s signal reflected a slower cGMP hydrolysis as a result of an incomplete PDE protein with limited catalytic capacity. To decipher more intricate details of channel or enzyme function in such a multiplexed setting, ratiometric calcium indicators could be used [24], although the variable number of AAV-borne CNG channels would skew the readings in each assay.

In conclusion, we developed a simple cell-based reporter based on calcium imaging that can identify potent AAV vectors with biological activity within 48 h. This in vitro model could serve high-throughput pipelines of novel gene therapy development and evaluate vector potency ahead of preclinical studies in vivo.

## 4. Materials and Methods

### 4.1. Cell Culture

Immortalized cells were cultured according to standard practices. Briefly, cells were cryopreserved in low glucose DMEM (11885084, Thermo Fisher, Darmstadt, Germany) supplemented with 20% FBS (S0615-500ML, Sigma-Aldrich, Taufkirchen, Germany) and 10% DMSO (C6295, Sigma-Aldrich) in liquid N2. To bring them in culture, cryovials were thawed and resuspended in warmed complete medium made of DMEM supplemented with 10% FBS and 1% Anti/Anti (15240062, Thermo Fisher). The cell suspension was centrifuged at 0.5 rcf for 5′ to remove the DMSO, the cell pellet was resuspended in complete medium and transferred to a sterile T-75 flask. Cells were maintained in an incubator at 37 °C, 5% CO_2_, 85–95% humidity and passaged once 90% confluent. For passaging, the medium was aspirated, the cells washed once with DPBS-CaCl_2_ and then detached from the flask using 0.05% Trypsin-EDTA (5′ incubation at 37 °C, 5% CO_2_). Cells were counted using 0.4% Trypan Blue Stain (T10282, Thermo Fisher) and the Countess3 FL Automated Cell Counter (Thermo Fisher) before seeding in a new recipient.

### 4.2. Generation of New Hela Cell Lines

The piggyBac vector was cloned using the backbone: pB09/TRE-MCS-SV40pA-Ef1a-rtTA-PuroR-SV40pA (kindly provided by Prof. Volker Busskamp, University of Bonn). The backbone was linearized via enzymatic digestion using Fast Digest (FD) BamHI (FD0054, Thermo Fisher) followed by FastAP Thermosensitive Alkaline Phosphatase (1 U/μL) treatment (EF0654, Thermo Fisher). The RhGC and GECI coding sequences were PCR-amplified from the RhGC(hBE)_pGEM (kindly provided by Prof. Peter Hegemann, Humboldt University of Berlin), pGP-CMV-GCaMP6s (Addgene #40753), pGP-CMV-NES-jRCaMP1a (Addgene #61562) and pGP-CMV-NES-jRCaMP1b (Addgene #63136) using Gibson Assembly primers. RhGC and GECI genes were linked by a T2A element, leading to a bicistronic transcript for simultaneous expression of both proteins in the presence of doxycycline. The resulting plasmids were: pB09/TRE-RhGC(BE)-T2A-GCaMP6s-SV40pA-Ef1a-rtTA-T2A-PuroR-SV40pA (11 kb), pB09/TRE-RhGC(BE)-T2A-jRCaMP1a-SV40pA-Ef1a-rtTA-T2A-PuroR-SV40pA (11.033 kb), pB09/TRE-RhGC(BE)-T2A-jRCaMP1b-SV40pA-Ef1a-rtTA-T2A-PuroR-SV40pA (11.033 kb). The puromycin resistance gene was used to select for HeLa cells in which successful genome integration had occurred. After transposition and a 7-day puromycin (1 mg/mL) selection period, single HeLa cells were picked, following a serial dilution step, and grown to separate colonies. Using 200 ng gDNA from the single colonies as template, quantitative PCR (qPCR) revealed which colonies had the highest number of inserts, and those were taken forward for all experiments.

### 4.3. Gene Expression Analysis

Extracted RNA from AAV-transduced cells or tissues was digested with RQ1 RNase-free DNase (M6101, Promega, Walldorf, Germany) to eliminate DNA contaminants. Fifty-200 ng of RNA were reverse-transcribed to cDNA using SuperScript IV Reverse Transcriptase (18090050, Thermo Fisher) or a RevertAid First Strand cDNA Synthesis Kit (K1621, Thermo Fisher). For all types of expression analyses, primer pairs were designed for eGFP, ITR2, GECIs, with an annealing temperature between 58 and 62 °C. Quantitative PCR was done using the PowerUp SYBR Green Master Mix (A25742, Thermo Fisher), according to the manufacturer’s instructions on the QuantStudio 5 Real-Time PCR platform.

### 4.4. RhGC Illumination and GECI Recordings

**Figure 6:**
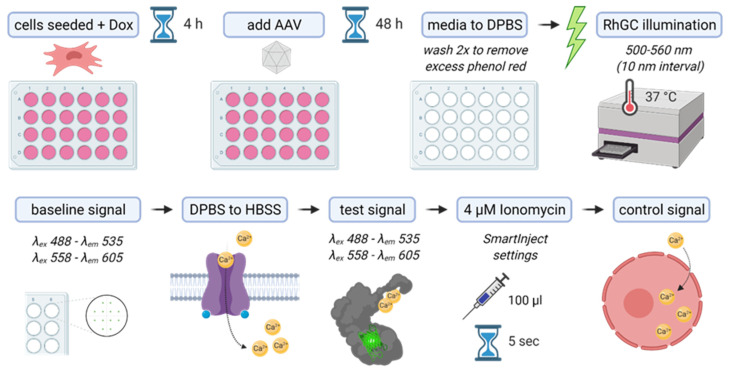
Illustration of the experimental design and set-up.

All illumination and fluorescence recordings were performed using the SpectraMax plate reader Multi-Mode Microplate Reader from Molecular Devices. The plate reader temperature was set at 37 °C before all recordings and the microinjector was loaded with an 8 μM ionomycin (I9657, Sigma-Aldrich) solution in HBSS^+CaCl_2_^ (14025092, Thermo Fisher). All recordings were performed in clear 24-well plates with signal reads performed from the bottom of the well with a high PMT and optics setting (Figure 6).

To account for discrepancies of cell distribution in each well, 12 dispersed points were recorded from each well and then averaged. Six technical repeats were performed for each condition. Cells were seeded in complete medium supplemented with doxycycline (0.5 μg/mL) and, once attached (~4 h), they were transduced with AAV vectors for 48 h. Ahead of GECI recordings in the plate reader, media were replaced by DPBS^−CaCl_2_^ (14190144, Thermo Fisher) and washed twice to ensure that residual phenol red was removed. The 24-well plate was then placed inside the pre-warmed (37 °C) plate reader and illuminated with a 500–560 nm spectrum at 10 nm intervals to activate RhGC. Consecutively, baseline GECI signal was recorded with single-wavelength settings; either λex 488–λem 535 for GCaMP6s or λex 558–λem 605 for jRCaMP1a and jRCaMP1b. DPBS^−CaCl_2_^ was replaced with HBSS^+CaCl_2_^, and then the test GECI signal was recorded using the same wavelength settings as before. With only 100 μL HBSS^+CaCl_2_^ covering the cells, the plate was reinserted in the plate reader and, using the SmartInject setting, 100 ul of 8 μM ionomycin were injected over the cells, shaken to mix (final concentration 4 μM), and after a 5 s pause, the control GECI signal was recorded.

### 4.5. Immunocytochemistry and Imaging

Cells were seeded on pretreated glass slides that were previously coated with poly-D-lysine (P6407, Sigma-Aldrich), according to the manufacturer’s instructions. Once attached, the cells were illuminated at the excitation wavelength (λex) of RhGC, i.e., 500–560 nm in 10 nm intervals, and then immediately fixed with 4% PFA/PBS for 10 min at room temperature. The cell monolayer was washed twice with PBS and then incubated overnight at 4 °C with primary antibodies diluted in blocking solution (2% BSA/0.3% Triton-X in PBS). The primary antibodies used were sheep anti-cGMP 1:3000 (from Prof. Steinbusch, Maastricht University, Maastricht, The Netherlands), rabbit anti-PDE6A IgG 1:500 (NBP1-87312, Novus Biologicals), rat anti-CNGA3 1:30 (custom-made anti-CNGA3, 7D8, hybridoma supernatant, kind gift of Heinz G. Körschen, Caesar Bonn). The next day, the cell monolayer was washed twice with PBS and incubated with a secondary antibody solution for 1.5 h at room temperature while covered. The secondary antibodies used were sheep anti-Alexa594 IgG (H + L), donkey 1:500 (A-11016 Thermo Fisher), anti-rabbit-Alexa488 IgG (H + L) 1:400 (#4412, Cell Signal), and anti-rat-Cy3 IgG (H + L) 1:200 (112-165-143, Jackson). Finally, the monolayer was washed once with PBS and incubated for 10 min with DAPI 1:2000 (D1306, Thermo Fisher) prior to mounting with a glass slide (18606-5, Aqua-Poly/Mount). Images were acquired with an inverted Leica SP8 confocal microscope. The original images, consisting of multiple z-stacks, were further processed with the open-source software Fiji [25].

### 4.6. AAV Vector Production and Cell Transduction

Vector production was performed as previously described [10,26]. Single-stranded AAV plasmids containing a mSWS-CNGA3, hArr3-CNGA3 or hRho-PDE6A expression cassette flanked by ITRs from AAV2 were used as vector plasmids. Genomic titers of AAV preps were determined by qPCR using primers: ITR2-F: 5′ ggaacccctagtgatggagtt 3′ and ITR2-R: 5′ cggcctcagtgagcga 3′. For transduction experiments, cells were passaged, counted, and seeded in a 50,000 cells/24-well plate density. Multiplicity of infection (MOI) was determined accordingly, and the calculated volume of AAV solution was added in the media over the cells and incubated for 48 h.

### 4.7. Statistical Analysis

Graphs and statistical analyses were performed using Prism 9 (Graph-Pad, San Diego, CA, USA). The results are presented either as paired data points across conditions or violin plots showing the distribution of all data points. Paired Student’s *t*-test was used to compare two conditions of the same sample population. The significance α = 0.05 was accepted and exact *p* values are recorded over each graph.

## Figures and Tables

**Table 1:** Primer sequences.

Target	Forward Primer	Reverse Primer	Annealing T °C
eGFP	CGACCACTACCAGCAGAACAC	TTCTCGTTGGGGTCTTTGCTCAG	62
ITR2	GGAACCCCTAGTGATGGAGTT	CGGCCTCAGTGAGCGA	58
GCaMP6s	CCCGACAACCACTACCTGAG	GTCCATGCCGAGAGTGATCC	60
jRCaMP1a	AAGACAGGTCACGCAGTCAG	GAGTGTAACCACGCAGACCA	60
jRCaMP1b	GGAATAAGTGGGGTCACGCA	GAGTGTAACCACGCAGACCA	60

## Data Availability

Not applicable.

## Supplementary Materials

The following are available online at https://www.mdpi.com/article/10.3390/ijms23094538/s1.
