# Supplementary material for: A Bioengineered In Vitro Model to Assess AAV-Based Gene Therapies for Cyclic GMP-Related Disorders
Source: Int J Mol Sci. 2022 Apr 20;23(9):4538. doi: 10.3390/ijms23094538 (PMC9101586; doi:10.3390/ijms23094538)
Supplement: Supplementary file 1 [file ijms-23-04538-s001.zip › ijms-1684893-supplementary.pdf]

Supplementary Materials:

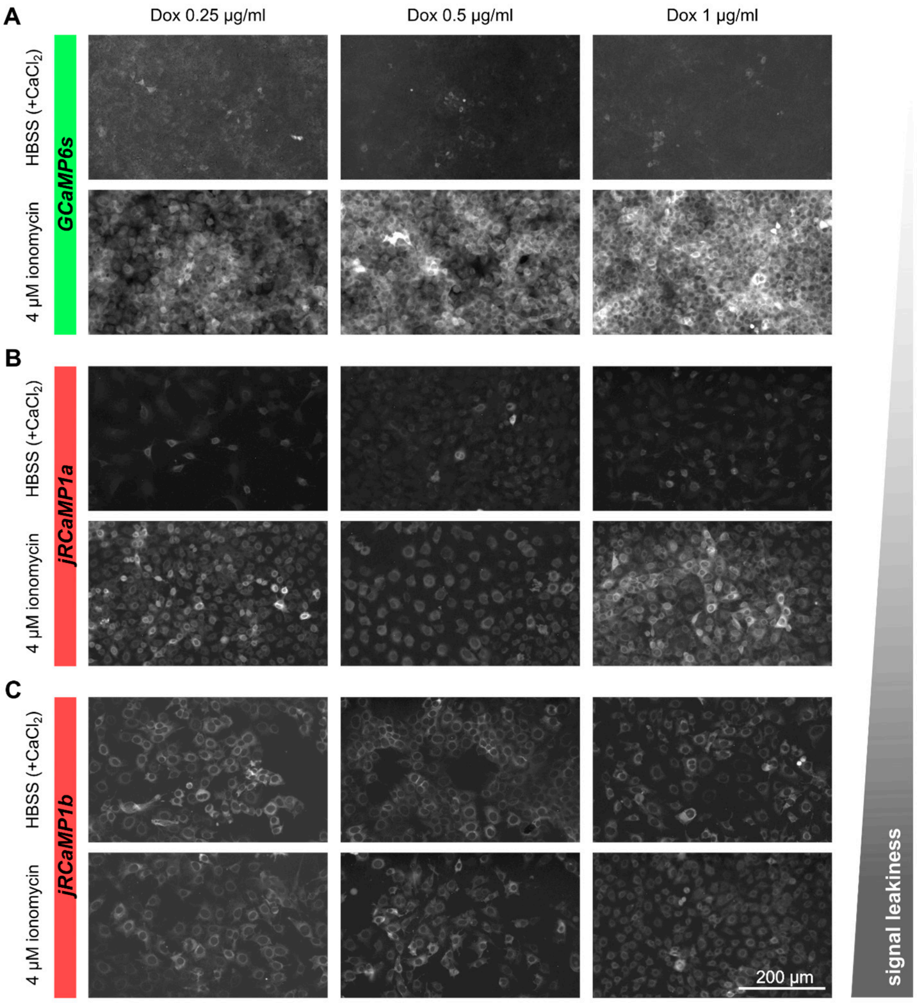

Supplementary Figure S1: *Assessment of doxycycline induced a Ca^{2+} signal in the HeLa cell lines engineered to express one of the three GECIs. (A) Representative fluorescence images of background GCaMP6s signal in HeLa cells exposed to increasing doxycycline concentrations (0.25-1 µg/ml) kept in a solution with high extracellular Ca2+ concentration (HBSS+CaCl2) versus positive control of GCaMP6s signal where cells are permeabilized with 4 µM ionomycin allowing for free diffusion and binding of Ca2+ to the intracellular GECI. (B-C) Same as in (A) for HeLa cells expressing jRCaMP1a and jRCaMP1b, respectively.*

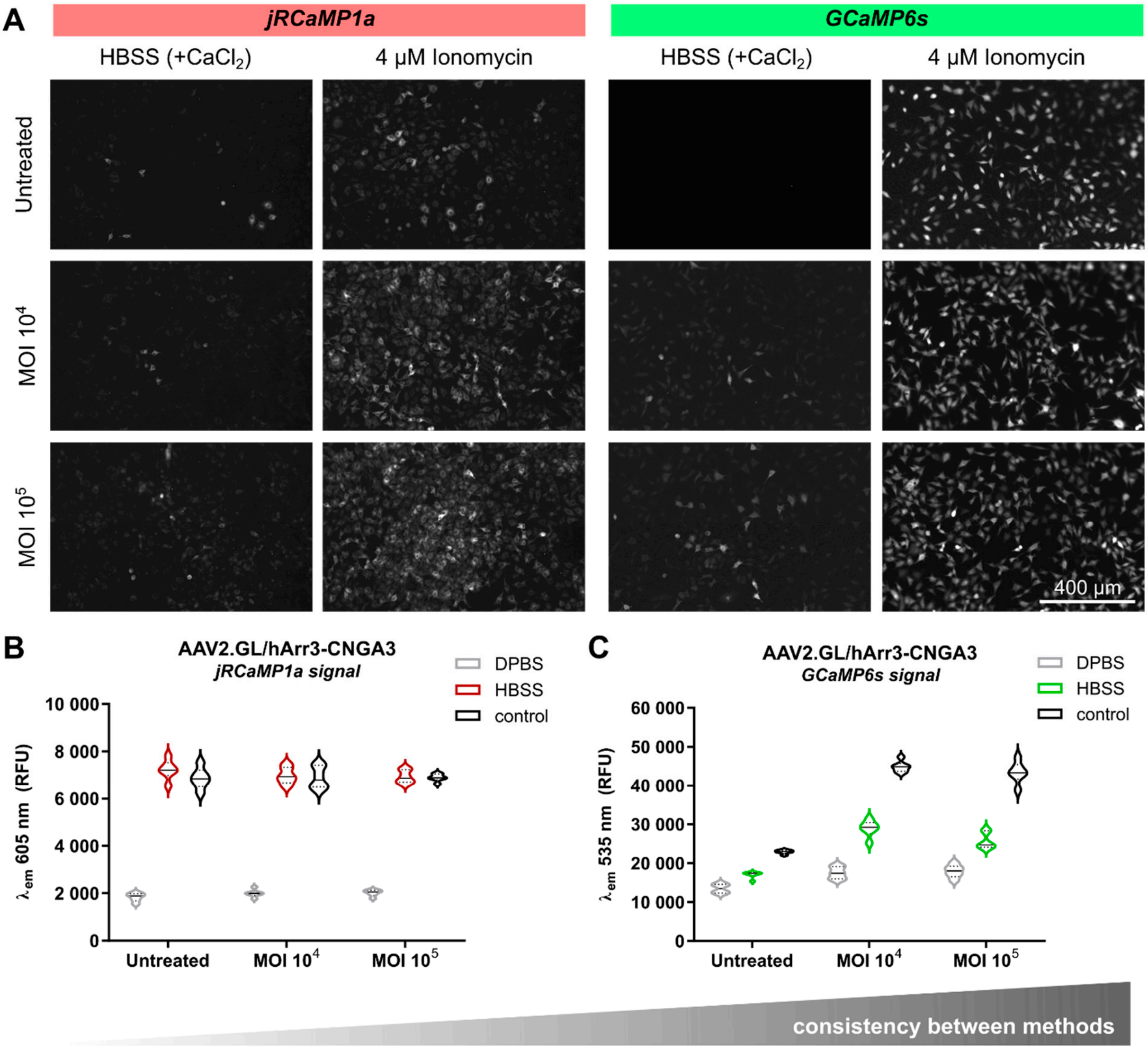

Supplementary Figure S2: *First proof-of-concept test using a novel AAV vector with CNGA3 cargo. (A) Fluorescence images of GECI signal obtained from engineered HeLa cells expressing RhGC-GECI and transduced with two MOIs of AAV.GL/hArr3-CNGA3, in the presence of high extracellular* Ca^{2+} *(*$HBSS^{+CaCl_2}$*) versus positive control signal where cells are permeabilized with a 4 µM ionomycin solution allowing the free diffusion and binding of* Ca^{2+} *to the GECI. (B) Quantification of relative fluorescence units (RFU) of engineered HeLa cells expressing RhGC-jRCaMP1a transduced with AAV2.GL/hArr3-CNGA3 in three consecutive conditions; in the absence of extracellular* Ca^{2+} *(*$DPBS^{-CaCl_2}$*), in high extracellular* Ca^{2+} *(*$HBSS^{+CaCl_2}$*), and positive control signal where cells are permeabilized with a 4 µM ionomycin solution. (C) As in (B) but for HeLa cells expressing RhGC-GCaMP6s. As evident from the comparison of the fluorescence images of the cells (A) with the summary data (B), jRCaMP1a showed less consistency in the observed changes in fluorescence signal, The consistency was higher with GCAMP6s, which prompted us to continue with this GECI.*

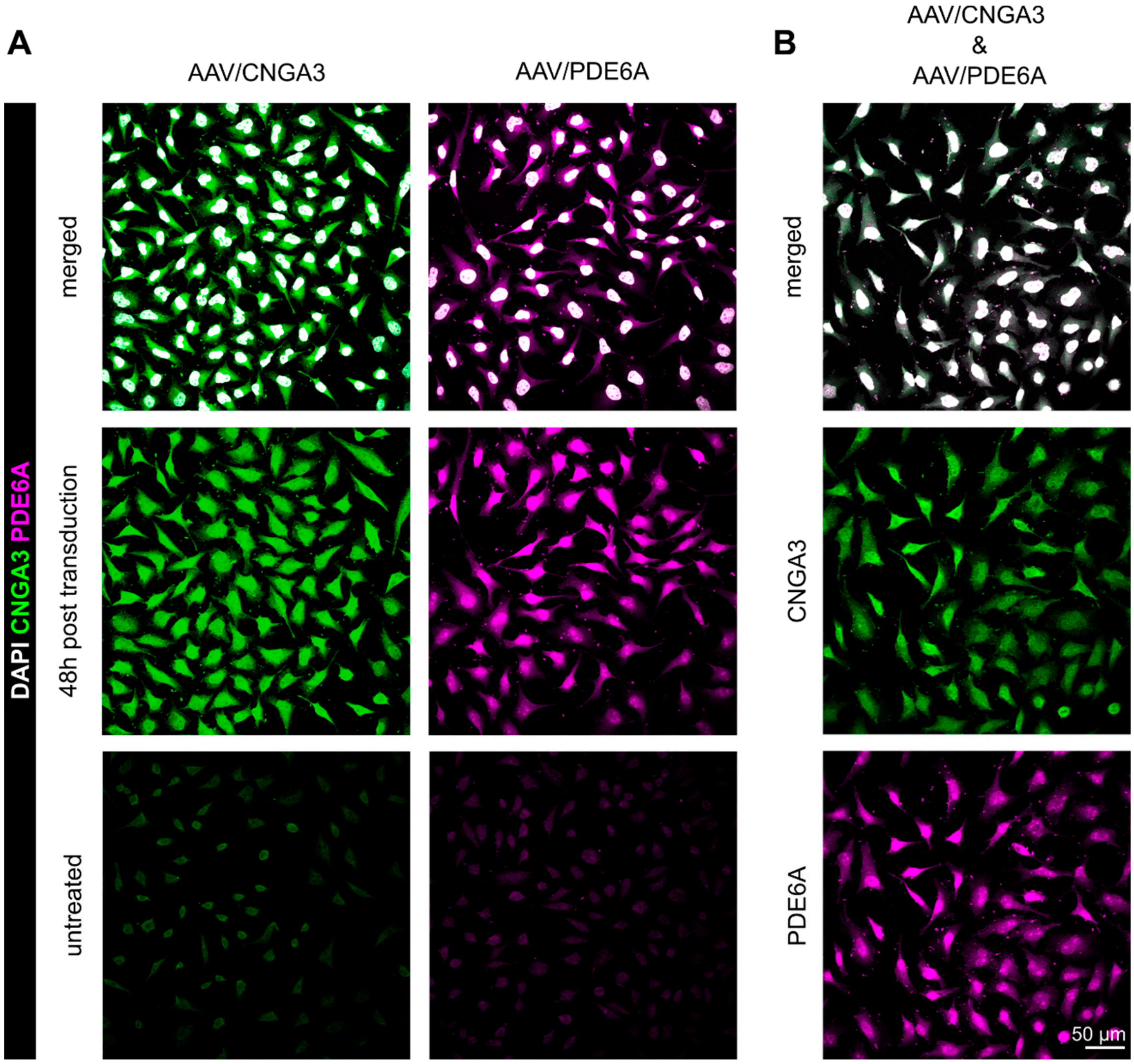

Supplementary Figure S3: *Evaluation of CNGA3 and PDE6A protein expression in HeLa cells after AAV transduction. (A) Confocal images of HeLa cells 48 hours post transduction with AAV2.GL delivering cargo hArr3-CNGA3 or hRho-PDE6A at MOI 10^4. CNGA3 protein is shown in green and PDE6A in magenta. Nuclei are stained with DAPI shown in white. The lowest panel shows minimal antibody background in untreated cells. (B) Confocal images of cells co-transduced with both AAV vectors at MOI 10^4 showing simultaneous CNGA3 (green) and PDE6A (magenta) staining. Nuclei are stained with DAPI (white).*
